# DNA methylation signature in peripheral blood reveals distinct characteristics of human X chromosome numerical aberrations

**DOI:** 10.1186/s13148-015-0112-2

**Published:** 2015-07-28

**Authors:** Amit Sharma, Muhammad Ahmer Jamil, Nicole Nuesgen, Felix Schreiner, Lutz Priebe, Per Hoffmann, Stefan Herns, Markus M. Nöthen, Holger Fröhlich, Johannes Oldenburg, Joachim Woelfle, Osman El-Maarri

**Affiliations:** Institute of Experimental Hematology and Transfusion Medicine, University of Bonn, Sigmund-Freud Str. 25, 53127 Bonn, Germany; Pediatric Endocrinology Division, Children’s Hospital, University of Bonn, Bonn, Germany; Institute of Human Genetics, University of Bonn, Bonn, Germany; Department of Genomics, Life & Brain Center, University of Bonn, Bonn, Germany; Institute for Computer Science, c/o Bonn-Aachen International Center for IT, Algorithmic Bioinformatics, University of Bonn, Dahlmannstr. 2, 53113 Bonn, Germany

**Keywords:** Klinefelter, Turner, DNA methylation, PAR region, X chromosome inactivation, Epigenetics

## Abstract

**Background:**

Abnormal sex chromosome numbers in humans are observed in Turner (45,X) and Klinefelter (47,XXY) syndromes. Both syndromes are associated with several clinical phenotypes, whose molecular mechanisms are obscure, and show a range of inter-individual penetrance. In order to understand the effect of abnormal numbers of X chromosome on the methylome and its correlation to the variable clinical phenotype, we performed a genome-wide methylation analysis using MeDIP and Illumina’s Infinium assay on individuals with four karyotypes: 45,X, 46,XY, 46,XX, and 47,XXY.

**Results:**

DNA methylation changes were widespread on all autosomal chromosomes in 45,X and in 47,XXY individuals, with Turner individuals presenting five times more affected loci. Differentially methylated CpGs, in most cases, have intermediate methylation levels and tend to occur outside CpG islands, especially in individuals with Turner syndrome. The X inactivation process appears to be less effective in Klinefelter syndrome as methylation on the X was decreased compared to normal female samples. In a large number of individuals, we verified several loci by pyrosequencing and observed only weak inter-loci correlations between the verified regions. This suggests a certain stochastic/random contribution to the methylation changes at each locus. Interestingly, methylation patterns on some PAR2 loci differ between male and Turner syndrome individuals and between female and Klinefelter syndrome individuals, which possibly contributed to this distinguished and unique autosomal methylation patterns in Turner and Klinefelter syndrome individuals.

**Conclusions:**

The presented data clearly show that gain or loss of an X chromosome results in different epigenetic effects, which are not necessary opposite.

**Electronic supplementary material:**

The online version of this article (doi:10.1186/s13148-015-0112-2) contains supplementary material, which is available to authorized users.

## Background

In mammals, the X chromosome has very unique characteristics: the presence of two X chromosomes in females and only one in males requires the existence of a dosage compensation mechanism(s) to ensure “nearly” equal genetic contributions in both sexes [1]. However, gene expression levels on the X chromosome still differ between males and females; this is due to unequal X inactivation across the X chromosome. Some genes escape the inactivation process [2], while other regions—specifically those with homology on the Y chromosome (termed pseudo autosomal regions)—are not inactivated (like on autosomes) in either 46,XY or 46,XX [3]. Therefore, an abnormal number of X chromosomes—loss of an X chromosome in females (Turner syndrome) or gain of an extra chromosome in males (Klinefelter syndrome)—can lead to profound imbalances in gene dosage on the X chromosome and thus to non-standard phenotypes in humans [4].

Sex chromosome abnormalities have a high prevalence in humans, with Klinefelter syndrome (47,XXY) being most common in men (1/500) and Turner syndrome (45,X) most common in women (1/2500) [5]. Such chromosomal abnormalities lead to a high risk of miscarriage and stillbirth. However, if an individual with Turner syndrome survives birth, they possess characteristic features, such as short stature, webbed neck and ovarian dysfunction, leading to infertility, osteoporosis, cardiovascular malformations, diabetes mellitus, and thyroid autoimmune diseases [6]. Klinefelter syndrome, on the other hand, apart from phenotypic features, such as high stature, gynecomastia, and infertility, is often linked to declined verbal skills [7]. Both syndromes have the hallmark characteristic of abnormal endocrine balance and display hypergonadotropic hypogonadism [8–10]. The details of the underlying molecular mechanisms that lead to the development of these symptoms associated with X chromosome abnormalities are poorly understood. In particular, molecular insights on epigenetic changes responsible for the occurrence of these phenotypes are missing.

Molecular studies to gain knowledge on the changes in epigenetics and gene expressions associated with abnormal numbers of chromosomes could improve the understanding of the molecular basis of these symptoms. To date, no systematic detailed studies on expression as well as epigenetic changes that combine both the gain (as in 47,XXY) and the loss (as in 45,X) of the X chromosome have been published. However, this issue is partially addressed by several papers that analyzed limited number of samples from a single available tissue. For instance, Klinefelter syndrome, a combined genome-wide expression and DNA methylation in one schizophrenic brain sample, was reported [11], as well as genome-wide expression analysis from testis tissue [12] or whole blood [13, 14]. Several other studies analyzed single loci in order to develop a molecular diagnosis method for detection [15, 16]. Regarding Turner syndrome, here, too, only few studies exist which performed genome-wide expression analysis, i.e., in fibroblast [17] and in cell free DNA derived form amniotic fluid [18]. In other studies, single loci were analyzed mainly for diagnostic purposes [19].

The relationship between the number of X chromosomes and clinical phenotypes can better be revealed when samples of gains as well as losses of an X chromosome are studied simultaneously under the same experimental conditions. For this reason, we studied the DNA methylation status of CpG islands and promoters in blood samples derived from Turner (45,X: a loss of one X) and Klinefelter (47,XXY: gain of one X) syndrome individuals and compared them to healthy males (46,XY) and females (46,XX).

Our results show that loss of an X chromosome has much more profound consequences than gain of an additional X chromosome. About five times more loci (considering only autosomal CpG sites) are affected in Turner than in Klinefelter individuals. Moreover, about 80 % of the changes in Turner individuals are hypomethylated, while nearly equal numbers of hypo- and hypermethylated CpG sites were observed in Klinefelter individuals. Affected loci, especially in Turner individuals, are characterized by intermediate methylation level in 46,XY males/46,XX females and are typically located outside of CpG islands. Methylation on the inactive X in Klinefelter samples appears to be less than what is observed in normal female samples, indicating a non-optimal inactivation process in Klinefelter individuals. Gene ontology analysis reveals clusters of genes, which are expected to be involved in the clinical phenotype. In Turner individuals, these were immune response, immune system processes, and meiosis, while in Klinefelter individuals, these included cellular processes involved in reproduction. We also highlight the association between haploinsufficient conditions and extent of the methylation state at pseudoautosomal regions (mainly PAR2) in X chromosomal aberrations.

## Results

### Illumina 27K genome-wide DNA methylation profiling reveals extensive methylation changes in both Turner and Klinefelter samples across the whole genome

**Fig. 1:**
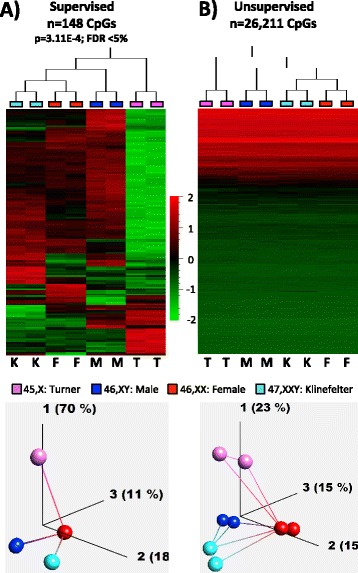
Hierarchical clustering and principal component analysis (PCA) of the Illumina 27K data. The figure is based only on autosomal loci. **a** Supervised analysis testing for differences between groups using ANOVA and applying a cutoff of <5 % false discovery rate (FDR), corresponding to a *p* value of 3.11E-4 and 148 CpG sites; **b** Unsupervised hierarchical clustering on the normalized data without applying a statistical hypothesis. The *heat maps* in the upper part of the figure correspond to one sample for each column and one locus for each horizontal line; *red* to *green* corresponds to relatively hypermethylated (*red*) to hypomethylated (*green*). In the lower part of the figure, each of the spheres corresponds to one sample and the connecting lines correspond to the two nearest neighbors (more similar) of a particular sample

**Fig. 2:**
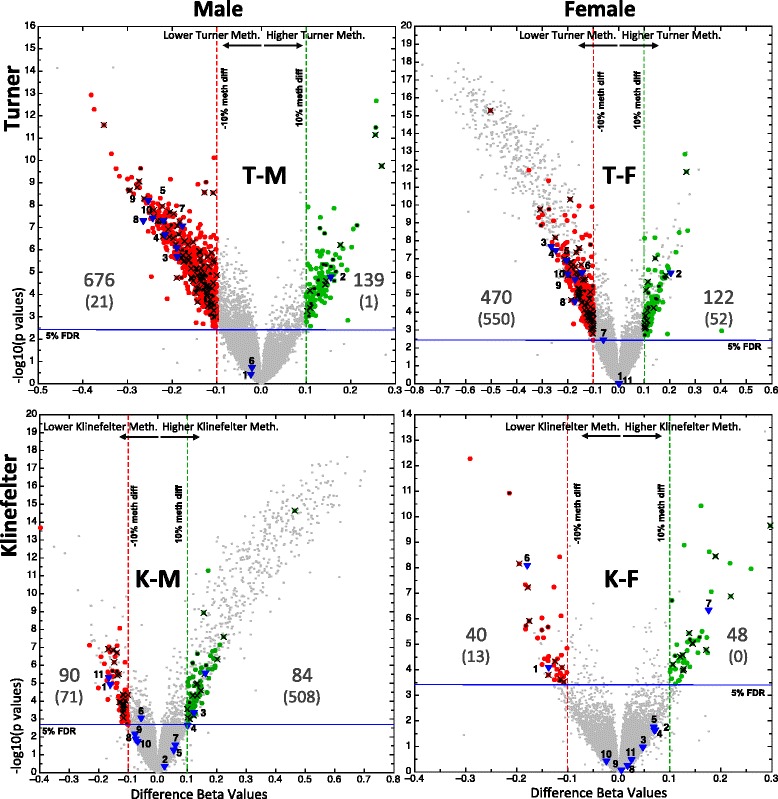
Volcano plots of the Illumina 27K data showing pairwise comparisons of Turner and Klinefelter samples to male and female samples. The differences in beta values between the respective comparisons is on the X-axis, while the –log10 (*p* values) is on the Y-axis. *Vertical dashed red/green* and *horizontal blue lines* represent the 10 % methylation differences and the 5 % false discovery rates cutoff, respectively. The differentially methylated autosomal CpGs (>10 % differences and FDR <5 %) are represented by *red* and *green filled circles* for hypo and hyper methylated loci respectively; their corresponding number is also shown. The verified CpGs by pyrosequencing are represented as *blue triangles* (1 = cg2487174, 2 = cg16848873, 3 = cg24169822, 4 = cg11418559, 5 = cg20191453, 6 = cg09697795, 7 = cg18059933, 8 = cg26306976, 9 = cg04451770, 10 = cg06812844, 11 = cg04452095; loci labels are as in Fig. 5). Cross-reactive loci, as defined by Chen Y. et al, [54] are labeled with a black “x”. The numbers in each figure correspond to the number of individual CpG sites on autosomes that are hypomethylated (*left*) and hypermethylated (*right*); below each number (*in parenthesis*) is the corresponding number of differentially methylated CpG cites on the X chromosome

Our pilot study (performed on Illumina 27K methylation arrays) showed considerable differences in methylation between Turner and Klinefelter individuals on the one hand and males and females on the other hand. When considering only the autosomal loci, a clear distinction of all four groups can be seen as Euclidean-based hierarchical clustering, while PCA analysis shows Turner samples to be the most distant from other samples (Fig. 1). The volcano plot in Fig. 2 illustrates the number of affected CpGs and the magnitude of differences. Turner samples harbored nearly five times more changes than Klinefelter samples. For this analysis, we define differential methylation as the differences of more than 10 % methylation (calculated as the difference between beta value transformed average *M* values—see Methods) and a false discovery rate of <5 %. On autosomes, 815 (676 hypo- and 139 hypermethylated in Turner samples) and 174 (90 hypo- and 84 hypermethylated in Klinefelter samples) different CpG sites are differentially methylated when comparing male to Turner and to Klinefelter samples, respectively. When comparing female to Turner and Klinefelter samples, 592 (470 hypo- and 122 hypermethylated in Turner samples) and 88 (40 hypo- and 48 hypermethylated in Klinefelter samples) CpGs are differentially methylated, respectively (for detailed list of affected genes see Additional file 1). On the X chromosome, a large number of differences were observed when a sample with one X was compared to another sample of two X, as in the comparison of Turner samples to female samples and male samples to Klinefelter samples. Far weaker differences were observed between Turner samples and male samples and between Klinefelter samples and female samples. This indicates that in Turner and Klinefelter individuals, X chromosome activity depends on the number of X chromosomes. This in turn suggests that the X chromosome in Turner individuals is as active as it is in males and that the second X in Klinefelter individuals is as inactivated as it is in females. Moreover, the differentially methylated regions in Turner individuals (on autosomes) have four to five times higher tendencies to be losing rather than gaining methylation.

### Characteristics of the differentially methylated regions

#### Methylation levels of affected CpGs

**Fig. 3:**
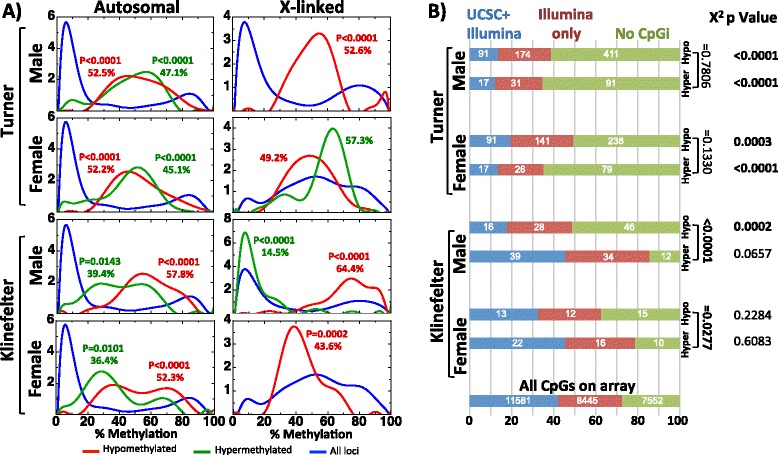
Characteristics of the differentially methylated CpGs. **a** The level of methylation of differentially methylated regions (*red* and *green curves* represent the normal methylation frequency of the hypomethylated and the hypermethylated CpG sites, respectively) in comparison to their methylation levels in male and female samples (*blue curves* represent the frequency of methylation distribution of all analyzed CpG sites). The *p* values of the Chi-square test for trend between the methylation frequency distribution of either the hypo- or hypermethylated loci in comparison to all CpG sites on the array are also shown (when significant), together with the average methylation. The autosomal (*left column*) and X-linked loci (*right column*) were analyzed separately. **b** CpG island status: the differentially methylated regions were investigated for their CpG island status and were divided into three groups, blue if the CpG occurs in a CpG island based on both the Illumina array annotation and the UCSC, red if the CpG occurs in a CpG island based on only Illumina array annotation, and green if the CpG occurs in no CpG island. Next, this data of each comparison is compared to the distribution of all CpGs in the array, and Chi-square *p* values are shown at the right. Also, *p* values of the comparison between the hypo- and hyper-locations are shown

The differentially methylated regions between each Turner and Klinefelter sample and each male and female sample were divided into eight groups as defined by the volcano plots in Fig. 2 (i.e., four comparisons resulting in hypo- (red, left side of the volcano plot) as well as hypermethylation (green, right side of the volcano plot)). Next, we studied the distribution of the methylation values at the affected CpGs of each of the eight groups in the male and female samples. The first obvious observations were that most of the affected loci have a medium level of methylation values. This was more pronounced in affected loci in Turner samples (Fig. 3a). Moreover, in Turner samples, the distribution of beta values of the hypermethylated sites (Av. = 46.1 %) and the hypomethylated sites (Av. = 52.3 %) are nearly overlapping and are approximately normally distributed with peaks at 40–60 %. In contrast, in Klinefelter samples (Fig. 3a), the two curves corresponding to hypo- (Av. = 55 %) and hypermethylations (Av. = 37.9 %) have shifted to the right and left, respectively. The latter suggests that in Klinefelter samples, the hypomethylated loci are relatively good methylated in male and female samples (i.e., >50 %) and that the hypermethylated loci tend to be relatively less methylated in male and female samples (i.e., <50 %). A chi-square trend test revealed significant differences of the beta value distribution between all eight groups (on autosomes) and the total CpGs on the array.

#### CpG islands status of the differentially methylated CpGs

In order to further understand the characteristics of the affected CpGs, we investigated whether the affected CpGs are located within CpG islands or not. In Turner samples, the affected CpGs are overrepresented outside CpG islands (Fig. 3b). This applied to the differentially methylated CpGs in comparisons to male and female samples. Equally, no difference in distribution was observed between the loci that were hypo- and hypermethylated in comparison to male and female samples. In Klinefelter samples, statistically significant differences were only observed for loci being hypomethylated when compared to male samples. These loci—as in Turner samples—were enriched outside CpG islands. At the affected CpGs in Klinefelter samples, we detected statistically significant differences in the CpGi status distribution between hypo- and hypermethylated CpGs.

#### The differentially methylated regions are largely not overlapping with age sensitive loci

Since we performed only two arrays hybridization per karyotype group and because the ages of the three used samples were not exactly the same (Turners, 13 ± 2 years; males, 18.3 ± 0.6; females, 18.3 ± 0.6; Klinefelters, 14 ± 2.6), we investigated whether the differentially methylated CpGs contain age sensitive loci. We compared our data with previous studies [20–22] that identified age sensitive CpGs mainly on the Illumina 27K. When comparing the four lists of differentially methylated regions (each of Turner and Klinefelter samples to each of male and female samples) to the lists of age sensitive CpGs, found in the three abovementioned studies, we detected little overlaps ranging from 0 to 3.4 % (Additional file 2). From this analysis, we concluded that the slight age difference between the samples did not contribute to enrichment of CpG sites that show a strong correlation with age. We also concluded that the differentially methylated regions in Turner and Klinefelter samples are generally not overlapping with age sensitive CpGs.

### Overlaps in affected loci between Turner and Klinefelter samples

**Fig. 4:**
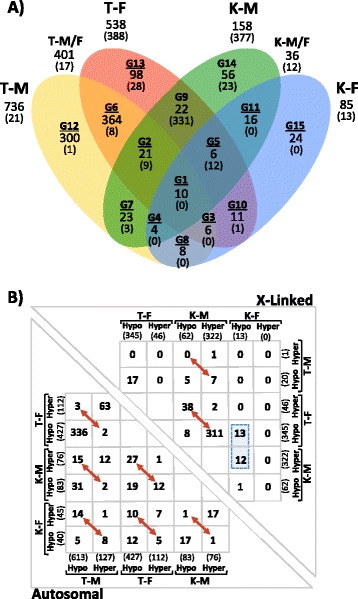
Overlaps in affected loci between Turner and Klinefelter samples. **a** Venn diagram showing the intersections between four comparisons. The comparisons are abbreviated as T-M, T-F, K-M, and K-F, where *M* male, *F* female, *T* Turner, *K* Klinefelter. Different intersection groups are labeled from G1 to G15. Below each group name the total number of genes is given as well as the number of genes on the X chromosome (*in parenthesis*). **b** Relative direction of methylation changes between the four different comparisons. The X-linked (*upper right*) and autosomal (*lower left*) loci are shown separately. The numbers in the Table correspond to the common genes between the X- and the Y-axis categories. The *red two-headed arrows* point to the gene numbers that switched orientation in their methylation changes. *Blue dashed boxes* highlight the X-linked genes that are hypomethylated in Klinefelter samples in comparison to female samples, suggesting weaker X inactivation in Klinefelter than in female samples

We then investigated whether the same genes/loci are affected in Turner and in Klinefelter samples. The Venn diagram in Fig. 4a shows that some overlap exists between the differentially methylated regions in Turner and Klinefelter samples. When considering the intersections of all four comparisons on autosomes, we observed only ten genes (Additional file 3) that are affected in all comparisons (G1). When comparing pairwise overlaps between Turner and male samples, Klinefelter and male samples, Turner and female samples jointly to Klinefelter and male samples and Klinefelter and female samples (G9, G5, G10, G3, G8, G4, G7, G2, G1), we observed 111 common genes. However, the majority of the affected 858 loci are non-overlapping loci (G12, G6, G13, G14, G11, and G15). This indicates a considerable degree of specificity of the methylation changes in Turner or Klinefelter individuals.

When considering the intersection between the differentially methylated regions in Turner samples on the one hand and male and female samples on the other hand, we noted that a good fraction of the affected loci are common (401 autosomal loci: G6, G2, G1, and G3), whereas in Klinefelter samples, the overlaps of differentially methylated genes with male and female samples are relatively few (36 autosomal loci: G11, G5, G1, and G4). This again indicates some degree of sex specificity in the differences.

We then investigated whether the directions of changes (i.e., hyper- or hypomethylation) are the same when comparing Turner or Klinefelter samples to either male or female samples. This was indeed the case for the vast majority of such loci. When comparing Turner samples to male (T-M) and to female samples (T-F), we observed 336 autosomal genes were hypomethylated (in Turner samples), while 63 genes were hypermethylated in comparison to both genders (Fig. 4b, lower left; Additional file 4). Only 5 loci showed an opposite direction of methylation in comparison to both genders (indicated by red arrows in Fig. 4b). In Klinefelter samples, the same situation was observed with 17 loci which were hypomethylated compared to male as well as female samples (K-M and K-F); 17 loci were hypermethylated and only 2 loci showed an opposite direction of methylation compared to both genders. In contrast to before, when comparing K-M to T-M and K-F to T-F, a considerable amount of loci showed opposite directions of methylation. Hence, some loci that were hypomethylated in Turner samples compared to male samples are hypermethylated in Klinefelter samples and vice versa. Regarding X-linked loci (Fig. 4b, upper right; Additional file 4), a small number of loci (13 X-linked genes) are hypomethylated in K-F and T-F, while at the same time hypermethylated in K-M. This suggests a weaker inactivation at these loci in Klinefelter individuals (indicated as blue dashed box in Fig. 4b).

### Gene ontology analysis matches the general clinical profile of Turner and Klinefelter syndromes

Although we did not analyze the gene expression in this study, we used the list of genes whose promoters showed differences in methylation of more than 10 % to investigate gene ontology enrichment. As this analysis will not necessarily show the genes that are affected in term of expression, it only shed more light on the function of genes whose promoters are >10 % differentially methylated. To avoid any background interference of methylation changes due to X inactivation, only autosomal loci were included in this study.

Turner samples showed a strong enrichment of immune-related processes (Additional files 5 and 6). This is true for the autosomal differentially methylated genes when compared to male and female samples. Specifically, the loci that differ between Turner and female samples included immune system processes, immune response, defense response to bacteria, cytokine production, and meiosis. These enriched categories reveal a clear link to the known relevant phenotype in Turner individuals, who are prone to frequent middle ear and renal infections [23–27]; furthermore, they are known to have higher rates of autoimmune disorders [28] and to be generally infertile [29]. In fact, the category meiosis contains fertility relevant genes, such as *DDX4* (DEAD (Asp-Glu-Ala-Asp) box polypeptide 4), which is specifically expressed in female germ cells [30], *TRIP13* (Thyroid Hormone Receptor Interactor 13), whose deficiency leads to loss of oocytes around birth [31], and *BRDT* (Bromodomain testis-specific protein), which is also expressed in mice oocytes [32]. Similarity in scatterplots [33] of the enriched gene ontology terms visualize unique categories and show that the “immune”-related terms present the highest enrichment with the lowest *p* values (Additional file 6: bigger blue circles).

No highly significant enrichment was observed for differentially methylated regions in Klinefelter samples (Additional file 5). This could be due to the relatively small number of affected genes. Only two terms were enriched when differentially methylated loci between Klinefelter and female samples were analyzed: (1) piRNA metabolic process and (2) positive regulation of keratinocytes proliferation. The two enriched GO categories against male samples were the following: (1) negative regulation of hydrogen peroxide metabolic process and (2) cellular process involved in reproduction in multicellular organism. The latter is in line with the known infertility associated with this syndrome. The respective GO category contains eight genes, some of which have been described to be directly involved in male infertility. For example, *PIWIL1* (piwi-like RNA-mediated gene silencing 1) plays a central role in spermatogenesis and was found to be differentially methylated in infertile patients [34], while lack of *SYCP1* (synaptonemal complex protein 1) in otherwise healthy mice has been found to yield infertility [35]. Polymorphisms in *MOV10L1* have been associated with azoospermic male infertility [36]. *MOV10L1* has been described to play an important role in the protection of spermatocytes against retrotransposons by piwi-interacting RNAs [37]. *GAPDHS* (glyceraldehyde-3-phosphate dehydrogenase, spermatogenic) is required for male mice fertility and sperm motility [38]. *SPACA1* deficient mice are infertile and show abnormally shaped sperm heads [39].

### Accurate pyrosequencing-based methylation measurement at selected autosomal loci confirms the array data

**Fig. 5:**
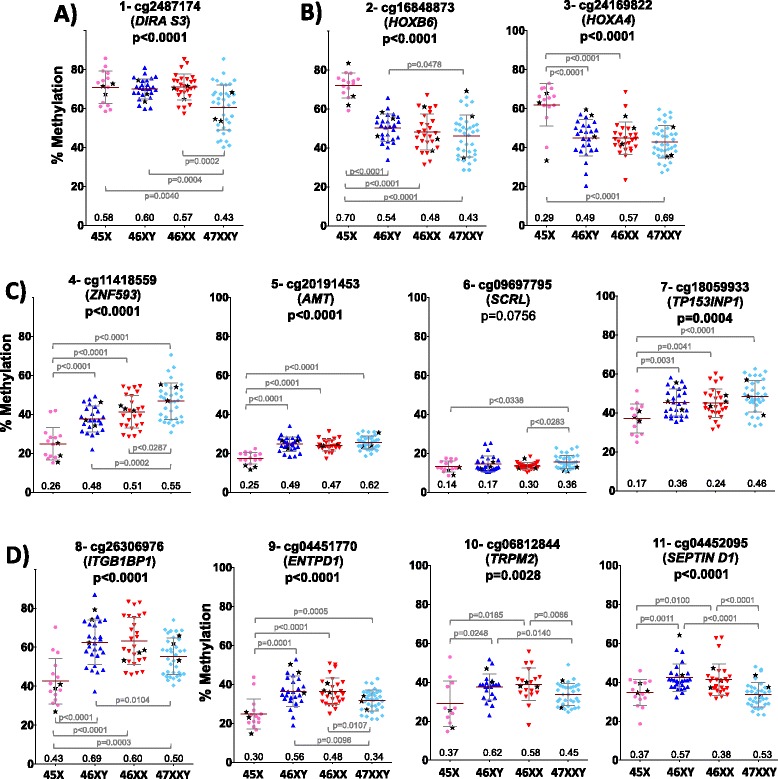
Detailed methylation data at selected loci in a large number of samples. The data at each locus, corresponding to 22 Turner (*pink filled circles*), 28 male (*blue triangles*), 28 female (*red inverted triangles*), and 40 Klinefelter samples (*sky blue diamonds*), are represented as vertical scatter plots with means (*red horizontal bars*) and standard deviation (*grey horizontal bars*). The *black filled stars* represent the samples used for the pool of DNA in the methylation array experiments. **a** Hypomethylated loci in Klinefelter samples, **b** hypermethylated loci in Turner samples, **c** hypomethylated in Turner and hypermethylated in Klinefelter samples, and **d** hypomethylated in Turner as well as Klinefelter samples. The Illumina ID number is shown at the top of each plot followed by the genetic name of the loci that appear in parenthesis and then by the *p* values of Kruskal-Wallis test for multiple group comparison; the *p* values of individual comparisons when significant are shown in *grey*

In order to verify the array data with an alternative method and a large number of samples, we selected several loci based on the differential methylation data from the 27K Illumina assay. Thus, we selected loci where only Klinefelter (Fig. 5a) or Turner samples (Fig. 5b) were significantly different compared to all others or where both Turner and Klinefelter samples were significantly different compared to male and female samples (Fig. 5c–d). For this analysis, we used pyrosequencing assays on all available samples from the four genotypes.

The results confirm the array data and clearly show that all samples within one genotype tend to be affected. At the same time, variability of methylation at most studied loci even in the male and the female samples was noted. Indeed, the range of methylation (in one genotype) increases to 40 % at some loci, and only three of the tested loci show relatively low variability (*AMT* and *SCRL*). This is in agreement with the above observations that the affected loci tend to be in the intermediate range of methylation and at weak or non-CpG islands, which are characteristics of CpG sites of variable methylation or CpG poor promoters [40].

While we did not include loci that show strong correlation with age (as shown in Additional file 2) in the above confirmation study, we nevertheless investigated the correlation between age and methylation for the loci studied by pyrosequencing (Additional file 7). We observed several nominal correlations (*p* < 0.05), but only 3 out (2 in males and 1 in Klinefelter) of 80 comparisons remained significant after Bonferroni correction for multiple testing. Therefore, age is also not a major factor influencing the methylation differences between the four karyotype groups at the pyrosequencing-verified loci. The individual loci-specific data was also used in hierarchical cluster and PCA analysis, demonstrating the ability of methylation markers to cluster different groups with perfect separation in one entity for Turner and male samples, while Klinefelter and female samples appear to be closer to each other (Additional file 8).

In order to investigate whether the methylation changes induced by lack or addition of one X are coordinated across the genome in a given individual, we studied the inter-loci correlations within every sample group, if there is indeed a strong stimulus that induces the methylation differences across all loci and if this stimulus is acting equally than we would expect strong inter-loci correlations in Turner and Klinefelter samples and to a higher degree than those observed in male and female samples. However, no widespread strong correlation was observed in any of the four groups (Additional file 9), indicating that the observed variations in both Turner and Klinefelter samples are not strongly coordinated genome-wide. However, once a locus is affected, the degree of variation could be stochastic in nature. Of interest is the relatively higher frequency of negative correlations between *DIRA S3*, *HOXB6*, and *HOXA4* with different loci in all groups, not just in Turner samples. This is unexpected because *HOXB6* and *HOXA4* are clearly hypermethylated (relative to male and female samples) only in Turner samples, while other loci show clear relative hypomethylation. The other groups did not show such clear methylation differences, suggesting that the observed changes in Turner samples display the same basic intrinsic tendency found in other genotypes but are somehow amplified by a still unknown mechanism in the 45,X genome.

### DNA methylation on the X chromosome reveals slightly lower methylation levels in Klinefelter individuals relative to females, significantly at the strongly inactivated loci, while Turner individuals show hypomethylation, relative to males at some PAR2 regions

**Fig. 6:**
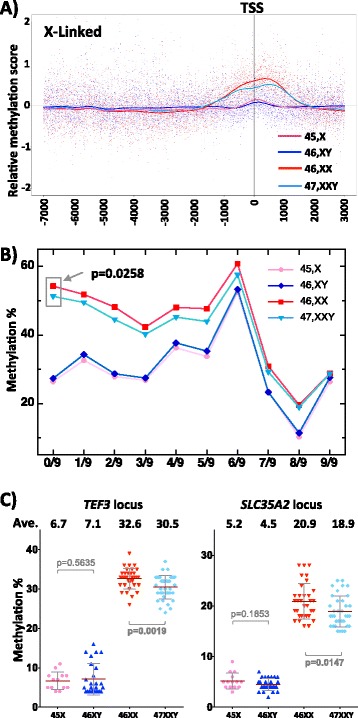
Trend in methylation differences at X chromosome between 46,XX and 47,XXY. **a** MeDIP data of the X chromosome in all four groups; all data are aligned to the transcription start site (TSS): 7 kb upstream and 3 kb downstream. **b** Illumina 27K methylation values; the data are divided into 10 pins depending on the degree of X inactivation of the corresponding locus (according to Carrel and Willard [2]); average methylation at each pin is represented in the plot and the significant *p* value between female and Klinefelter samples at 0/9 is shown; all other comparisons between either female and Klinefelter or between male and Turners were not significant. **c** Detailed pyrosequencing data on two X-linked loci (*TEF3* and *SLC35A2*) showing the differences in average methylation between female and Klinefelter samples

**Fig. 7:**
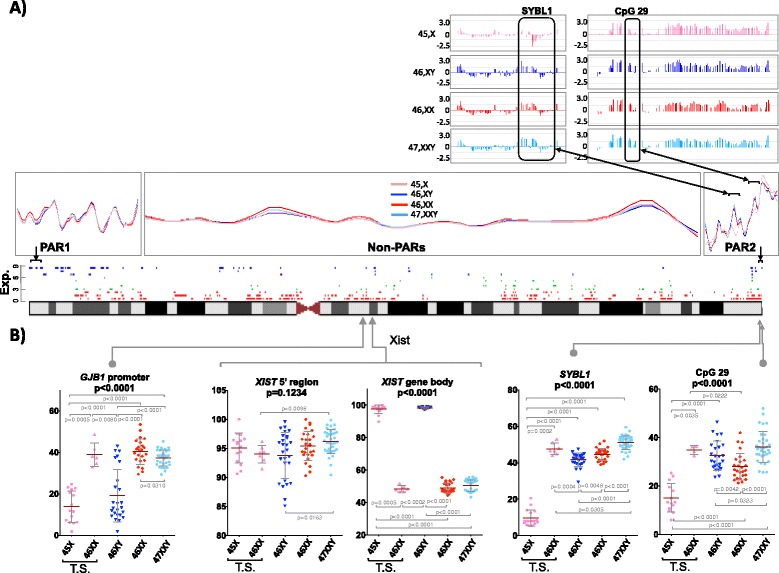
Methylation at PAR regions. **a** MeDIP-based data. *Lower panel* shows the scatter plot with smooth curve fitted by Loess (without gaps). Three different parts separately represent the PAR1, PAR2 and the non-PAR region. The *upper panel* highlights the raw MeDIP data as shown by the SignalMap software for the loci that were also studied by pyrosequencing (shown in part B of the Figure). **b** Pyrosequencing at two loci in PAR2: *SYBL1* and CpG29. The *p* values of Kruskal-Wallis test for multiple group comparison are shown below the name of the locus; the Mann-Whitney *p* values of individual comparisons when significant are shown in *grey*. In the middle of the figure, a diagram of the X chromosome is displayed together with the transcripts and their level of expression (on a scale of 0 to 9 to the left of the figure). *T.S* Turner syndrome

As abnormal numbers of sex chromosomes have shown a drift of methylation at autosomes, they might also influence the X chromosome inactivation status. Therefore, we performed thorough methylation analysis of the X chromosome by MeDIP followed by tilling array-based methylation analysis covering about 10 Kb around all promoter gene regions and all CpG islands including the PAR regions (that were not covered by the Illumina 27K), thus, enabling a study of global chromosomal changes. Particularly, when we aligned all studied promoter region in one sample to the transcription start site on the X chromosomes, we observed that the samples with one X (male and Turner samples) show similarly low methylation values, while the two samples with two X (female and Klinefelter samples) show relatively higher methylation levels consistent with the expected X inactivation-associated methylation on the inactive X chromosome (Fig. 6a). However, this analysis also revealed a clear relative hypomethylation of Klinefelter samples compared to female samples, suggesting that methylation at the inactivated loci on the additional X in Klinefelter samples is not as high as in female samples. This was further confirmed by the Illumina data where the difference in methylation was clearly more pronounced at the strongly inactivated regions (Mann-Whitney test *p* = 0.0258; group 0/9 according to Carrel and Willard [2]) and was not significant at either the middle inactivated or the escapees (groups 1 to 9; Fig. 6b). Furthermore, using the sensitive pyrosequencing method, we confirmed the existence of relative hypomethylation in Klinefelter samples compared to female samples at *TEF3* (*p* = 0.0019), *SLC35A2* (*p* = 0.0147) loci (Fig. 6c), and *GJB1* (*p* = 0.0310) (Fig. 7b).

The striking differences were more pronounced at the PAR2 region. PARs were not covered by the 27K Illumina array. However, several genes from PAR1 as well as PAR2 are covered by the MeDIP array analysis. The qualitative MeDIP data show that, indeed, differences exist regarding methylation status of X chromosomes, whereas mainly Turner samples showed obvious differences at PAR2 (Fig. 7a). We next analyzed the four groups of samples using pyrosequencing and, additionally, we included a group of seven Turner samples with the 46,XX karyotype (in the majority of cells) (the latter should undergo X inactivation) and investigated the methylation status of two loci at PAR2. The first analyzed locus was the *SYBL1* (*VAMP7*) at PAR2 that showed a distinguishable pattern of methylation. There was obvious methylation similar to methylation at X-inactivated CpG sites (i.e., about 50 % methylation) with the existence of two copies of the gene (either X + X or X + Y), whereas nearly no methylation was observed at 45,X Turner samples (Fig. 7b). Moreover, even a gradual increase of methylation was observed from 46,XY to 46,XX to 47,XXY, suggesting that methylation levels correlate with the copy numbers. This also agrees with a report showing that *SYBL1* (*VAMP7*) locus is inactivated on both the inactive X chromosome and the Y chromosome [41].

The 46,XX Turner samples were also as highly methylated as observed in female 46,XX samples (Fig. 7b). Interestingly, this methylation trend at the PAR2 region was not limited up to *SYBL1* genes. We observed similar behavior for the CpG29 island that lies at the telomeric end of PAR2. Therefore, based on the analysis of the selected genes of PARs, we hypothesized that PAR2 regions display unique behavior that distinguishes them from PAR1. Control regions (at non-PAR regions: *GJB1* and *XIST* gene body) subject to inactivation showed the expected methylation levels consistent with the number of X chromosomes (Fig. 7b). As part of our ongoing effort regarding X chromosomal abnormalities, we here provide DNA methylation of selective PAR2 regions as an independent assay for evaluation and close discrimination between unknown clinical genotypes.

## Discussion

Turner and Klinefelter syndromes are caused by loss or gain of one X chromosome (in the female and the male, respectively) [42]. Little is known about the molecular link between the abnormal X chromosome count and the phenotype. In this study, we aimed to contribute to the understanding of the molecular changes in these two syndromes. Toward this end, we analyzed DNA methylation in whole blood samples from these two syndromes as well as from healthy male and female individuals.

### X chromosome numerical abnormalities affect methylation on autosomes

We observed that methylation of mainly autosomal loci is affected in both Turner and Klinefelter syndromes. Most of them are found in the monosomic state of the X chromosome (45,X). Differences at autosomal genes, such the ones verified by pyrosequencing HOXA4, HOXB6, DIRAS3, ZNF593, AMT, SCRL, TP53INP1, ITGB1BP1, ENTPD1 TRPM2, and SEPTIN D1 (Fig. 5), clearly demonstrate the impact of sex chromosomal abnormalities. In case of the Turner phenotype, the hypermethylation status of *HOXA4* and *HOXB6* suggests a strong effect on developmentally important genes. Apart from that, genes involved in immune system processes, immune and defense response to bacteria, cytokine production, and meiosis were highly enriched in Turner samples (Additional file 6). This clearly emphasizes the validity of our findings because these are commonly expected syndromes in Turner individuals.

It is known that blood lymphocytes are central in adaptive immune responses. Thus, it not surprising that we could identify methylation signature enriched with major immune response factors from studying relevant blood samples in Turner individuals. However, surprisingly, we could also detect signatures of genes with altered methylation levels that are functional in tissues, which are developmentally far from blood like reproduction tissues/organs. Possibly, changes in methylation arise systematically in several or all tissues and are not restricted to only one specific tissue. Confirming our findings are two studies that performed genome-wide expression studies with Turner cells [17, 18]. The differentially expressed genes from amniotic fluid RNA reveal enrichment of expression in hematologic/immune cells [18], while from fibroblast, the enrichments were associated with biological functions, such as bone differentiation, glucose metabolism, and gonadal developmental pathways [17]. However, when comparing overlaps of autosomal differentially expressed loci from the above studies and our list of differentially methylated loci, only minor overlaps can be seen: *SERPINB10* and *LRRC17* (differentially expressed in fibroblast) [17] and *LEP*, *USP10*, *HPS4*, *SYNE2*, *PILRA*, *MST1*, *TRIP6*, *NPR2*, *CLEC2D* (differentially expressed in fibroblast) [18].

In analogy to Turner syndrome, Klinefelter samples only harbored minimal methylation changes. The relatively small number of affected loci was also observed by several recent studies [11–14]. As none of these studies analyzed methylation from whole blood, direct comparison to our data is difficult. However, there was no overlap in the top identified loci between all four studies, not even when we compare the study of Huang et al. [13] and Zitmann et al. [14], who both studied expression from the same tissue as we did, i.e., whole blood. Moreover, even when we compare our differential methylation data (autosomal loci in whole blood) to the differential methylation data from cerebellum and prefrontal cortex [11], we detected only 1/26 (*OR2L13*) and 2/23 (*TTC15* and *PIWIL1*) overlaps, respectively. This is not surprising, considering that (1) the comparison made from the same two individual brain tissues revealed only four overlaps (*RIPK1*, *PTPRN2*, *C8orf71*, and *SPAG1*) and (2) no overlap was detected between differentially expressed and differentially methylated loci. These disappointing comparison results could be due to a large inter-individual and/or inter-tissue variability that still need to be addressed in larger studies.

Moreover, the absence of global correlation between methylation and expression (in Turner and Klinefelter syndrome) are possibly due to the fact that the relatively small methylation changes (what we observed mostly in the range of 10–20 % of difference) represent a cellular memory; whereby, the cell still retains slight differential methylation at loci that were strongly differentially expressed during earlier stages of embryonic development. Such a “memorial epigenetic” signature was observed earlier in iPS cells derived by factor-based reprogramming from different tissues [43, 44]; as it was shown that methylation marks specific for the original cells could still persist in the produced iPS. Similar cases of methylation memory were reported for past EBV infections [45], for past smoking [46] or even for past pregnancy in mice mammary glands [47].

Meanwhile, the relatively small number of affected loci in Klinefelter (compared to Turner) samples may be due to very effective dosage compensation by inactivation of the additional X. Yet the question remains as to why the mechanism of dosage compensation remains different or less effective in case of Monosomy X (Turner), although the latter is the normal situation in males. The answer to this, most probably, is the presence of Y chromosome in males, which may harbor factor(s) that are needed for an appropriate genome-wide epigenetic balance and/or provide regions that pair with telomeric regions on the X (PAR1 and PAR2) and thus modulate expressions [48, 49]. Therefore, the expression of PAR1 (2.6 Mb size, 29 genes) and PAR2 (320 kb size, three genes) is modified on the X chromosome when comparing Turner samples to both male and female samples. Indeed, our data provide indirect evidence for this scenario since the methylation patterns of PAR2 differed dramatically between 45,X and 46,XY (Fig. 7).

### Klinefelter samples are significantly less methylated than female samples on strongly silenced loci on the X chromosome

The extra X chromosome in Klinefelter individuals forces the cell not only to strictly apply the rule of X inactivation, but also to face the interference of additional genes that escape from inactivation on the additional chromosome. Therefore, this female-like dosage compensation in the presence of a Y chromosome may lead to non-typical inactivation of the additional X in Klinefelter samples. Indeed, we observed significantly lower methylation on X loci than in female samples. Although the statistical significance was limited to strongly silenced genes (Fig. 6), the trend was observed even in moderately (or incompletely) inactivated loci (2/9 till 6/9; Fig. 6b). This was also reported in a previous study, which quantitatively determined methylation at few loci, in 47,XXY and 46,XX groups [16]. The *PGK1* strongly inactivated locus (upon X chromosome inactivation) showed statistically reduced methylation in the Klinefelter groups (56 % methylated in comparison to 69 % in females), while *FTHL17* that escapes the effect of inactivation still showed the tendency of being hypomethylated in 47,XXY (87 % methylated in comparison to 90 % in females). Furthermore, at the XIST locus, no statistical differences between Klinefelter and female samples were observed. However, a tendency to increased methylation in the XIST 5′ region and gene body was observed in Klinefelter samples (compared to female samples) in our data. Therefore, we speculate that a slightly higher methylation at promoter of XIST in Klinefelter individuals could lower the efficiency of inactivation (Fig. 7).

## Conclusions

In summary, our results reveal that X chromosome numerical variations affect methylation at large numbers of loci, mainly autosomal (Fig. 1). While specific DNA methylation-based biomarkers identified in our study can largely distinguish Turner and Klinefelter samples from female and male samples, respectively (Additional file 8), they can also be used for detailed molecular analysis of differential patterns of DNA methylation and epigenetic changes associated with aneuploidies (Fig. 2). On autosomes, male, female, and Klinefelter samples appear to be more or less similar in their methylation patterns, while Turner samples are distinguishably different (Figs. 1 and 2). Nonetheless, Turner samples are still closer to female than to male samples. Interestingly, methylation patterns on PAR regions (Fig. 7) appear to differ between male and Turner samples, and the inactivation of the second X chromosome in Klinefelter appears to be less effective than in females as could be inferred from the decreased methylation on the X-linked CpG sites (Fig. 6).

Our analysis is the first descriptive and comparative study of the DNA methylation status of X chromosome abnormal phenotypes under the same experimental conditions along with the comparison of healthy male (46,XY) and healthy female samples (46,XX). With the presented data it is possible to hypothesize that, although Klinefelter (46,XXY) and female samples (46,XX) undergo the process of X inactivation, the mechanism of X inactivation might vary somewhat between female and Klinefelter samples. Based on these data, we highlight the impact of X chromosomal aberrations on genome-wide methylation, which could contribute to sex disparities and disease development in Turner and Klinefelter syndrome individuals.

## Methods

### Patients, sample preparations, and ethics statement

Blood samples were collected from 25 Turner syndrome patients: 18 samples with 45,X as revealed by conventional haplotyping, while the remaining 7 samples consist of 2 with mosaic 45,X0/46,XX (80–95 % of peripheral blood cells consist of 46,XX) and five with 46,XX with a variety of structural abnormalities on the inactive X. All individuals show the classic clinical Turner phenotype. All samples were obtained from the Pediatric Endocrinology Division, University Children’s Hospital, Bonn, Germany. Forty Klinefelter syndrome patients (expected karyotype 47,XXY) were recruited to this study through the German Klinefelter Association, Falkenstein, Germany. In addition, control blood samples, from 28 healthy men and 28 healthy women with normal X chromosome count, were collected from blood donors at the Institute of Experimental Hematology and Transfusion Medicine, Bonn, Germany. All samples from all subjects were obtained upon written informed consent. The study was approved by the local ethics committee of the University Clinics of Bonn (approval numbers 106/05 and 121/06). In addition to conventional haplotyping, the X chromosome copy number from the used blood samples in all individuals was confirmed by two copy number assay as previously reported [50]. All samples were collected in Germany, and all individuals are apparently of German origin with the exception of two Turner and two Klinefelter individuals.

### DNA methylation analysis

#### Methylated DNA immunoprecipitation (MeDIP) analysis

For the MeDIP experiments, DNA derived from three individuals of the same karyotypes were equimolarilly mixed to reduce the effect of inter-individual methylation differences and further used for fragmentations. The samples used were chosen to be as close as possible in their age and of German origin (in years, 11, 13, and 15 years old for Turners (45,X); 18, 18, and 19 years old for males (46,XY); 18, 19, and 19 years old for females (46,XX); 11, 15, and 16 years old for Klinefelters (47,XXY)). DNA was fragmented by sonication (Branson Sonifier B-12: output control to microTip limit, 35 times at 5 s intervals); verification of the successful fragmentation in term of size and quantity was verified on 1 % agarose gels. Immunoprecipitation of methylated DNA in the fragmented DNA was done using the Methylated DNA IP kit from Zymo Research (catalog number D5101) according to the manufacturer’s instructions. To verify the enrichment of the methylated portion of DNA after immunoprecipitation, several real time quantitative TaqMan-based quantitative PCRs were used, namely CpG islands in UBE 2B (non-methylated), C21orf93 (methylated), LDH-C (methylated), and a no CpG control (data not shown). The immunoprecipitated DNA as well as the input DNA were further amplified using Sigma WGA2 kit. The amplified product was submitted to NimbleGen DNA Methylation Service, and the samples were hybridized to the 2.1 M Human Deluxe arrays that cover about 10 Kb of all promoters (from 7250 bases upstream of each TSS to 3250 bases downstream) and all CpG islands in hg18.

#### Illumina’s Infinium HumanMethylation27 array

The same DNA mixes used in the MeDIP experiments were also processed with Illumina’s Infinium HumanMethylation27 arrays. The bisulfite conversion of DNA was done using Zymo Gold kit; the Illumina array processing was performed according to standard protocols. Each sample was done in duplicate.

Raw data (beta values) was extracted from Illumina Bead Studio (version V2011.1). Methylation data was further processed via the Bioconductor lumi package [51], which works on *M* values rather than on beta values. The relationship between beta and *M* values is given as [52]:

$$ M={ \log}_2\frac{\beta }{1-\beta } $$

We used shift-scale color bias adjustment and quantile normalization as further preprocessing steps implemented in the lumi package. A detection *p* value cutoff of 0.00001 was used to filter out signals below background, and only CpGs in autosomes were considered. We used limma [53] to assess differential methylation of CpG sites between patient groups. Cross reactive loci were defined as described by Chen Y et al, [54]

#### Bisulfite pyrosequencing analysis

Genomic DNA (5 μg), extracted from blood of various genotypes was bisulfite converted using the EZ 96-DNA methylation kit (Zymo Research, Irvine, CA, USA) following the manufacturer’s standard protocol. Pyrosequencing assays were designed using the Pyromark Assay Design Software (Qiagen, Germany). Bisulfite PCR amplification was performed using Hot Star Taq DNA polymerase (Qiagen, Germany) and optimized cycling conditions. Pyrosequencing was performed using the Pyromark Q96 (Qiagen, Germany). A full list of bisulfite PCR and sequencing primers is given in Additional file 10.

#### Statistical analysis and data processing

Statistical analysis was done using the Prism software (GraphPad Software Inc.) or the SAS software (SAS for Windows, version 9.1; SAS Institute Inc., Cary, NC, USA). Principle component analysis, heat maps, and visualization of sample specific differences were done using the Qlucore Omics Explorer version 2.3 (Qlucore AB, Sweden). The MeDIP-array methylation data was normalized and visualized using R software.

#### Gene ontology analysis

Gene ontology enrichment analysis was done using the GOrilla server [55] and further visualization was done using REVIGO visualization tool [33].

## Additional files

Additional file 1:
**List of differentially methylated genes based on the Illumina 27K data.** The gene names in red are the loci studied by pyrosequencing and presented in Fig. 5; the number in parenthesis corresponds to the loci number in Fig. 5. Additional file 2:
**Venn diagrams showing the overlaps between differentially methylated CpGs and age sensitive DMRs.** The overlaps between differentially methylated regions from the four comparisons (each Turner and Klinefelter with each male and female) with age sensitive loci as determined by three studies. The list of Illumina ID cg numbers are listed for each intersection and the percentage of loci overlapping with age sensitive CpGs is given in red. Additional file 3:
**List of genes in the groups of loci presented in Fig.** 4a **representing intersections between different comparisons.** The gene names in red are the loci studied by pyrosequencing and presented in Fig. 5, the number in parenthesis corresponds to the loci number in Fig. 5. Additional file 4:
**List of genes in the groups of loci presented in Fig.** **4b** **.** The gene names in red are the loci studied by pyrosequencing and presented in Fig. 5, the number in parenthesis corresponds to the loci number in Fig. 5. Additional file 5:
**Summary of gene ontology anaylsis.** Details with functional ontology analysis of the differentially methylated loci in the four comparisons: each of the Turner and the Klinefelter samples with each of the male and the female samples. Additional file 6:
**Functional gene ontology analysis in Turner samples.** Scatter plots representing all non-overlapping functional categories are represented for both comparisons compared to male (left) and female samples (right). Bubble color indicates the user-provided *p* value (legend in upper right-hand corner) with the blue colored bubble being the most significant; size indicates the frequency of the GO term in the underlying GO database. X- and Y-axis represent two-dimensional space derived by applying multidimensional scaling to a matrix of the GO terms’ semantic similarities. The figures were generated with the REVIGO online tool [33]. Additional file 7:
**Spearman Correlation between age and pyrosequencing methylation results of individual loci.** Each column corresponds to one karyotype/group and each row corresponds to a locus. *p* values and Rho values are represented as heat maps on the left and write part respectively. The significances after Bonferroni correction for multiple testing are labeled with a black square. Additional file 8:
**Hierarchical clustering and PCA analysis of the single loci methylation pyrosequencing data.** A) The complete data, non-supervised analysis; B) at a 5 % FDR that left six loci on the list. Additional file 9:
**Correlation analysis between methylation data of individual loci studied by pyrosequencing, A) for individual CpGs, B) for the average of one region.** The loci included in this analysis cover the ones from Fig. 5 for autosomal loci and Fig. 7 for X-linked loci. Each of the four groups is represented in a separate heat map, whereby the upper right triangle represents the correlation *p* value and lower left triangle represent the Spearman Rho value. B, F and M: significance in male and female samples (B), only in female samples (F) and only in male samples (M); C: significance in healthy male and female controls. Additional file 10:
**List of primers.** All primers used for PCR amplifications and pyrosequencing.
