# Supplementary material for: DNA methylation signature in peripheral blood reveals distinct characteristics of human X chromosome numerical aberrations
Source: Clin Epigenetics. 2015 Jul 28;7(1):76. doi: 10.1186/s13148-015-0112-2 (PMC4517491; doi:10.1186/s13148-015-0112-2)
Supplement: Additional file 2: — Venn diagrams showing the overlaps between differentially methylated CpGs and age sensitive DMRs. The overlaps between differentially methylated regions from the four comparisons (each Turner and Klinefelter with each male and female) with age sensitive loci as determined by three studies. The list of Illumina ID cg numbers are listed for each intersection and the percentage of loci overlapping with age sensitive CpGs is given in red. [file 13148_2015_112_MOESM2_ESM.pdf]

Overlap between differentially methylated CpGs and age sensitive DMRs
(Autosomes only)

Bell et al, Plos Genetics, 2012
Illumina 27K data: 490 CpGs
Blood samples
172 female twins
32-80 years old

Age sensitive Set (490 CGs)

T-M (815 CGs) cg06268694 cg03544320 cg21870884 cg19594666 cg03355526 cg15747595 **0,7%**

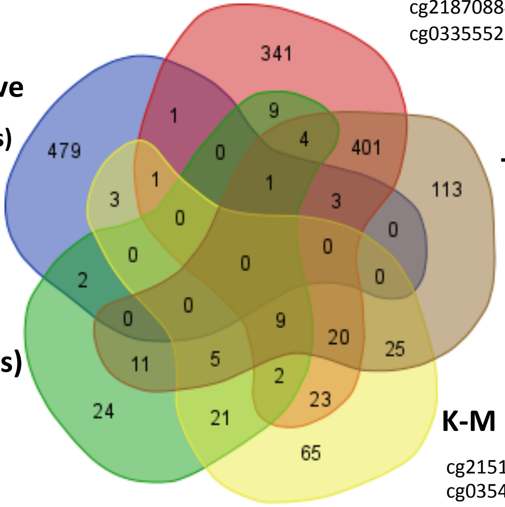

T-F (592 CGs) cg21870884 cg19594666 cg03355526 cg15747595 **0,7%**

K-F (88 CGs) **3,4%** cg03336167 cg04726200 cg19594666

K-M (174 CGs) cg21517055 cg03544320 cg12782180 cg19297232 **2,3%**

Rakyan et al, Genome research, 2010
Illumina 27K data: 131 CpGs
Blood samples and CD4+, CD14+
93 females
49-75 years old

Age sensitive Set (131 CGs)

T-M (815 CGs) cg18006568 cg21252483 cg10362475 cg19594666 cg03355526 cg15747595 cg22809047 cg08418111 **1%**

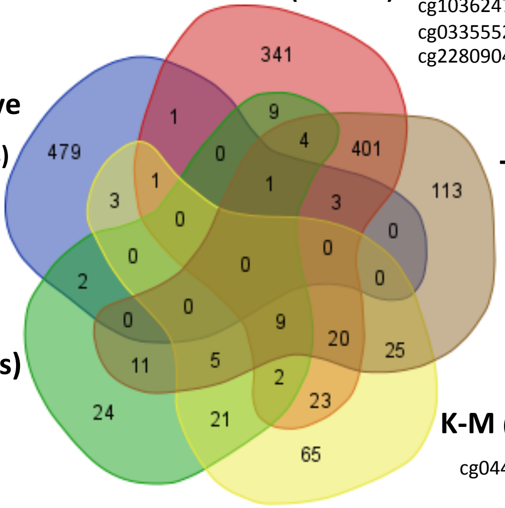

T-F (592 CGs) cg19594666 cg03355526 cg15747595 cg22809047 **0,7%**

K-F (88 CGs) **3,4%** cg03336167 cg04726200 cg19594666

K-M (174 CGs) cg04456238 **0,6%**

Weidner et al, Genome Biology, 2014
Illumina 27K data: 102 CpGs
Blood samples
0-73 years

Age sensitive Set (102 CGs)

T-M (815 CGs) cg00563932 cg16744741 cg03340878 cg17791651 cg21120249 cg10917602 cg17421623 cg20366832 cg21870884 cg15804973 cg04123409 cg26927807 cg09462576 cg23320649 cg26581729 cg20264732 cg23124451 **2,1%**

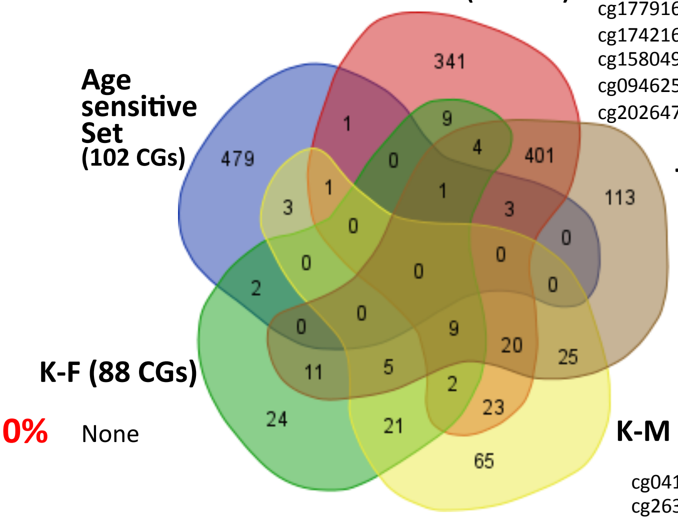

T-F (592 CGs) cg17791651 cg20366832 cg21870884 cg08598221 cg23320649 cg20264732 cg23124451 **1,2%**

K-F (88 CGs) **0%** None

K-M (174 CGs) cg04123409 cg26394940 **1,2%**
