# Supplementary material for: DNA methylation signature in peripheral blood reveals distinct characteristics of human X chromosome numerical aberrations
Source: Clin Epigenetics. 2015 Jul 28;7(1):76. doi: 10.1186/s13148-015-0112-2 (PMC4517491; doi:10.1186/s13148-015-0112-2)
Supplement: Additional file 6: — Functional gene ontology analysis in Turner samples. Scatter plots representing all non-overlapping functional categories are represented for both comparisons compared to male (left) and female samples (right). Bubble color indicates the user-provided p value (legend in upper right-hand corner) with the blue colored bubble being the most significant; size indicates the frequency of the GO term in the underlying GO database. X- and Y-axis represent two-dimensional space derived by applying multidimensional scaling to a matrix of the GO terms’ semantic similarities. The figures were generated with the REVIGO online tool [33]. [file 13148_2015_112_MOESM6_ESM.pdf]

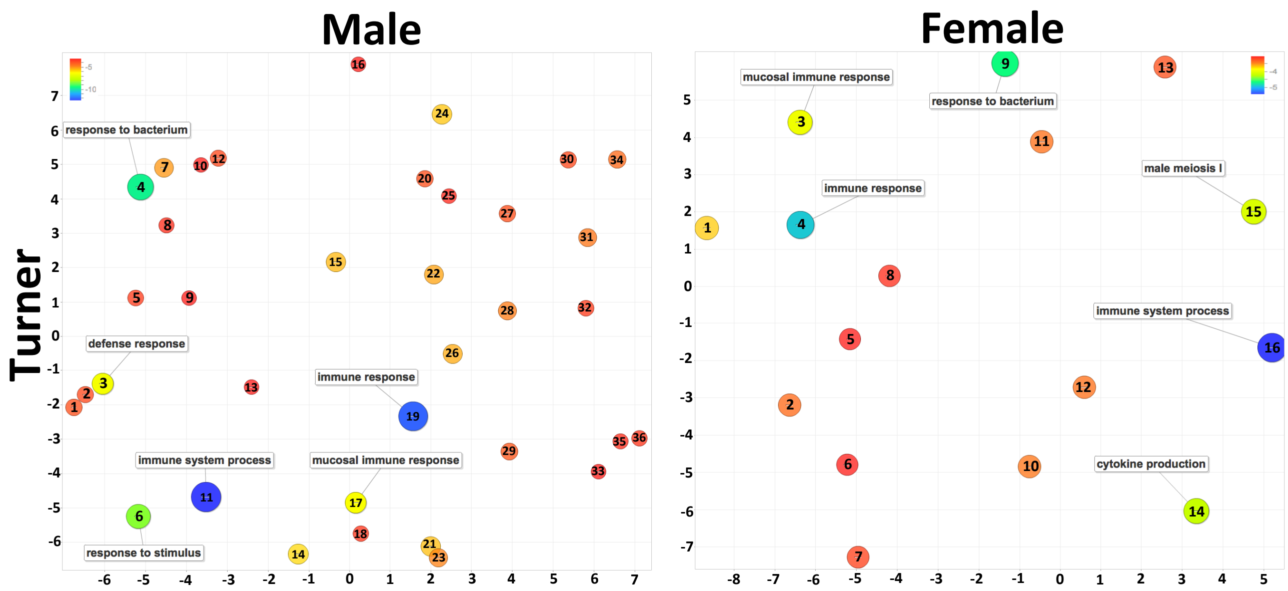

1-Acute inflammatory response
2-Inflammatory response
3-Defense response
4-Response to bacterium
5-Antibacterial humoral response
6-Response to stimulus
7-Kiling by host of symbiont cells
8-Antimicrobial humoral response
9-Cell Killing
10-Modification of morphology or physiology of other organism
11-Immune system process
12-Negative regulation of growth of symbiont in host
13-Reactive oxygen species metabolism
14-Organ or tissue specific immune response
15-Response to biotic stimulus
16-Cell activation
17-Mucosal immune response
18-Humoral immune response
19-Immune response
20-Regulation of response to stimulus
21-Myeloid leukocyte mediated immunity
22-Response to external stimulus
23-Neutophil mediated immunity
24-Regulation of cell adhesion
25-Cell surface receptor signaling pathway
26-Regulation of immune system process
27-Positive regulation of response to stimulus
28-Positive regulation of immune system process
29-Leukocyte migration
30-Regulation of interleukin-1 production
31-Positive regulation of cytokine secretion
32-Positive regulation of antigen processing and presentation
33-Chemotaxis
34-Cytokine production
35-Response to oxygen-containing compound
36-Drug transport

1-Organ or tissue specific immune response
2-Leukocyte migration
3-Mucosal immune response
4-Immune response
5-T cell receptor signaling pathway
6-Positive regulation of smooth muscle cell migration
7-Drug transport
8-Regulation of immune system process
9-Response to bacterium
10-Regulation of cell activation
11-Negative regulation of growth of symbiont in host
12-Regulation of cell-substrate adhesion
13-Integrin activation
14-Cytokine production
15-Male Meiosis I
16-Immune system process
