# Supplementary figures and images for: DNA methylation signature in peripheral blood reveals distinct characteristics of human X chromosome numerical aberrations
Source: Clin Epigenetics. 2015 Jul 28;7(1):76. doi: 10.1186/s13148-015-0112-2 (PMC4517491; doi:10.1186/s13148-015-0112-2)

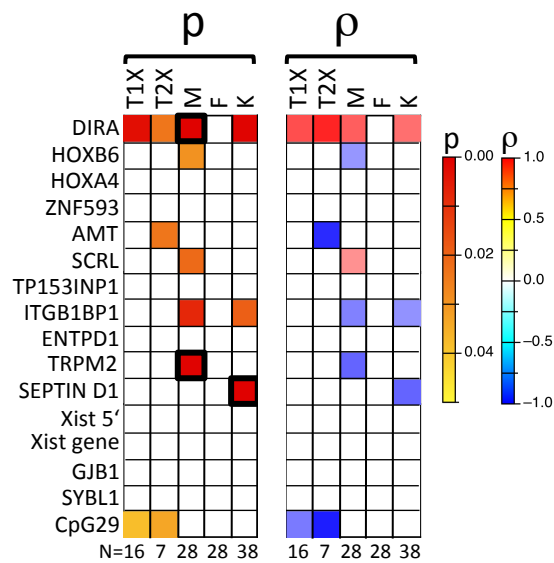

Significance after correction for multiple testing using a Bonferroni threshold of  $p < 0.000625$  (0.05/80)

Supplement: Additional file 7: — Spearman Correlation between age and pyrosequencing methylation results of individual loci. Each column corresponds to one karyotype/group and each row corresponds to a locus. p values and Rho values are represented as heat maps on the left and write part respectively. The significances after Bonferroni correction for multiple testing are labeled with a black square. [file 13148_2015_112_MOESM7_ESM.pdf]

**A)**

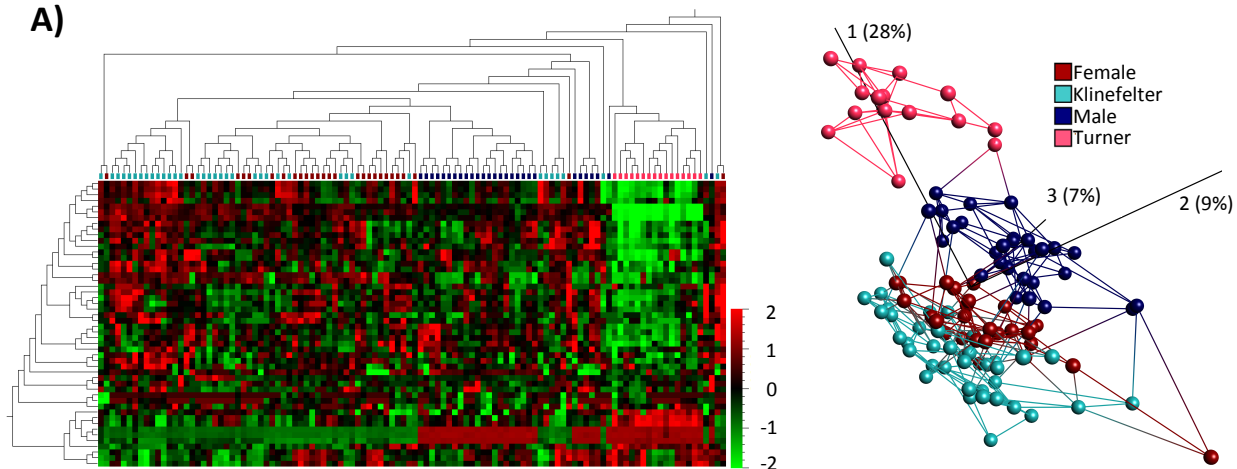

**B)**

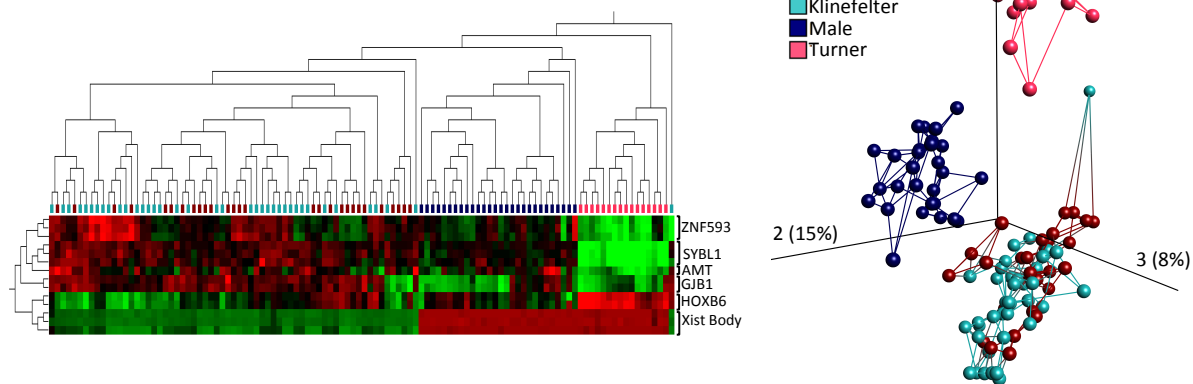

Supplement: Additional file 8: — Hierarchical clustering and PCA analysis of the single loci methylation pyrosequencing data. A) The complete data, non-supervised analysis; B) at a 5 % FDR that left six loci on the list. [file 13148_2015_112_MOESM8_ESM.pdf]

A)

Turner 45,X (n=16)

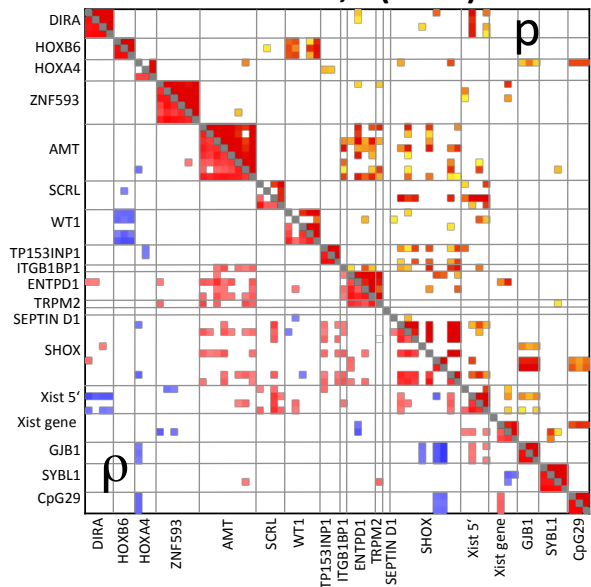

Klinefelter 47,XXY (n=38)

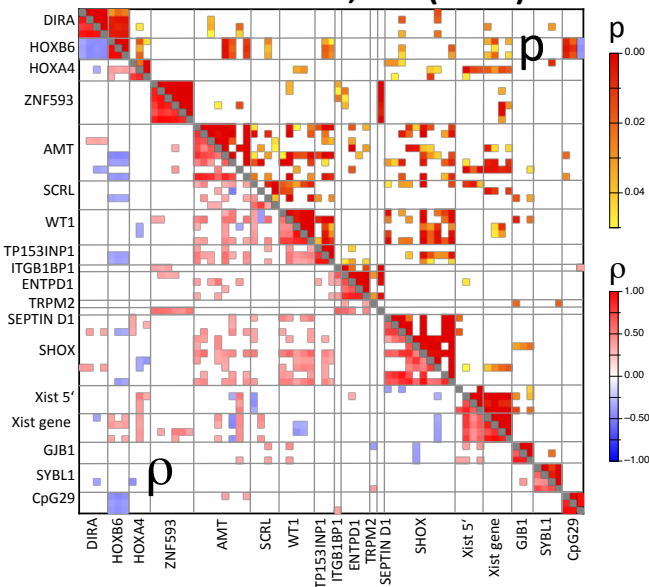

Male 46,XY (n=28)

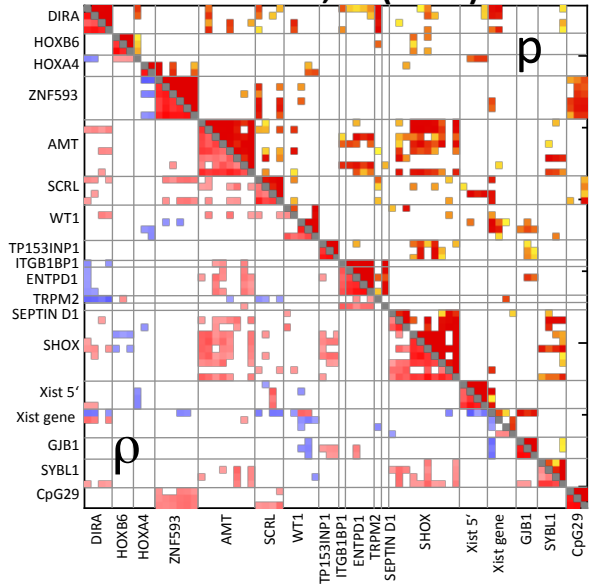

Female 46,XX (n=28)

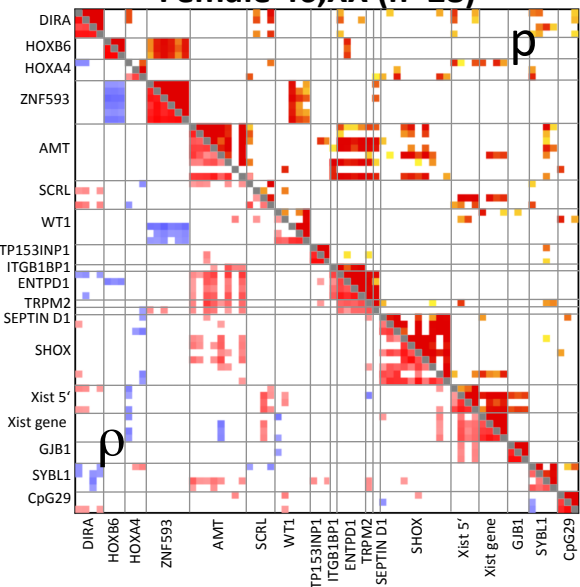

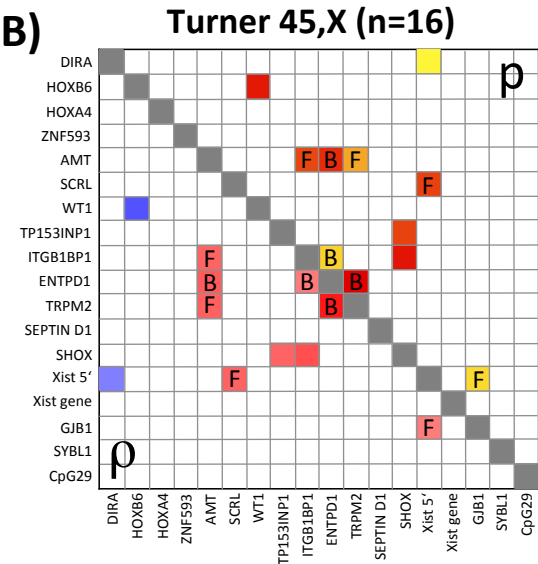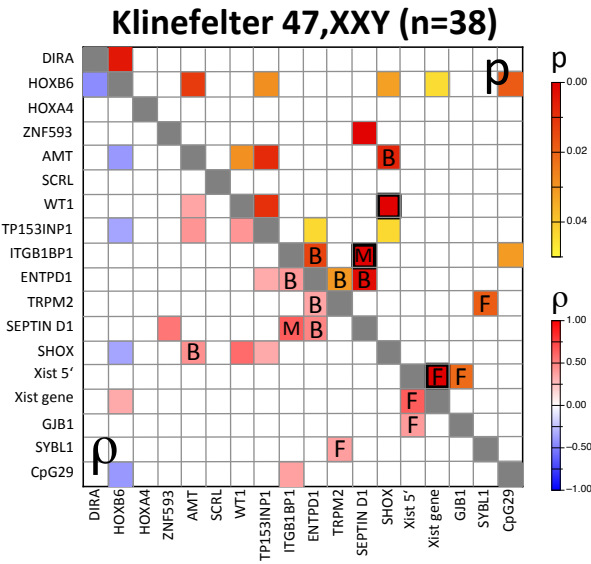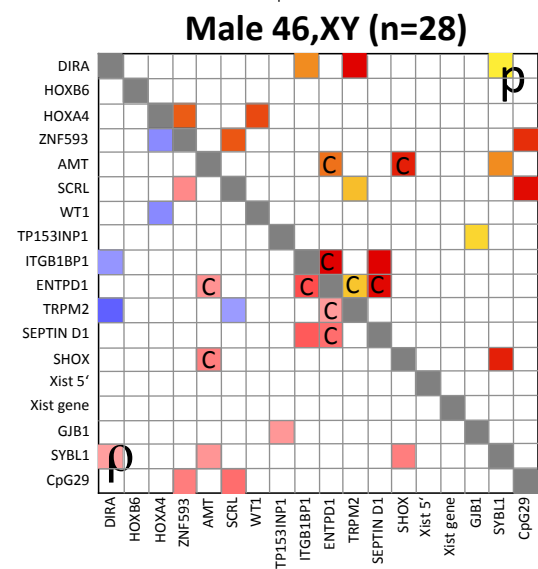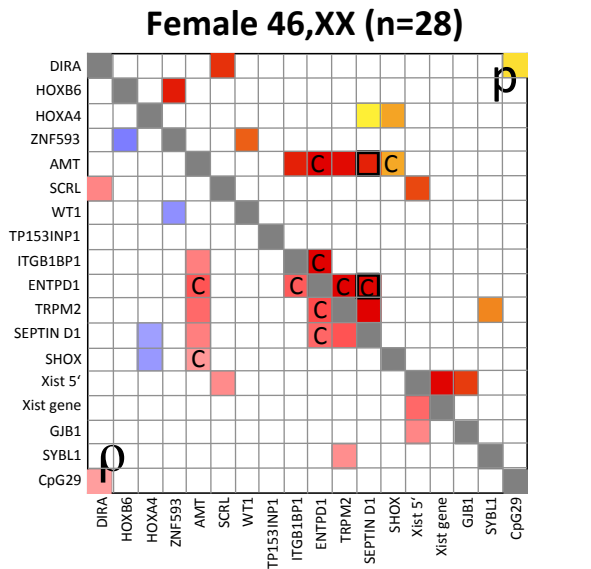

Supplement: Additional file 9: — Correlation analysis between methylation data of individual loci studied by pyrosequencing, A) for individual CpGs, B) for the average of one region. The loci included in this analysis cover the ones from Fig. 5 for autosomal loci and Fig. 7 for X-linked loci. Each of the four groups is represented in a separate heat map, whereby the upper right triangle represents the correlation p value and lower left triangle represent the Spearman Rho value. B, F and M: significance in male and female samples (B), only in female samples (F) and only in male samples (M); C: significance in healthy male and female controls. [file 13148_2015_112_MOESM9_ESM.pdf]
